# Oolong tea polysaccharide and polyphenols prevent obesity development in Sprague–Dawley rats

**DOI:** 10.29219/fnr.v62.1599

**Published:** 2018-12-19

**Authors:** Tao Wu, Jinling Xu, Yijun Chen, Rui Liu, Min Zhang

**Affiliations:** 1 Beijing Advanced Innovation Center for Food Nutrition and Human Health, Beijing Technology and Business University, Beijing, China; 2 State Key Laboratory of Food Nutrition and Safety, Tianjin University of Science and Technology, Tianjin, China; 3 Tianjin Food Safety & Low Carbon Manufacturing Collaborative Innovation Center, Tianjin, China

**Keywords:** oolong tea, anti-obesity, polysaccharide, polyphenols

## Abstract

**Background:**

Several studies have evaluated the effects of oolong tea extracts on obesity. However, only few studies focused on the anti-obesity effect of specific components of oolong tea.

**Objective:**

This study investigated the specific anti-obesity capabilities of oolong tea polysaccharide (TPS) and tea polyphenols (TPP) in high-fat diet-induced Sprague–Dawley rats.

**Methods:**

Oolong tea water extract, TPS, TPP, and polysaccharide mixed with polyphenol (TPSM) given at doses of 400 or 800 mg/kg were administered to rats fed with high-fat diet for 6 weeks to explore the anti-obesity effects of the treatments.

**Results:**

TPS and TPP were responsible for the suppressive effect on body fat accumulation. TPSM exhibited the highest effect on body weight reduction, and TPS and TPP effectively reduced serum leptin levels and significantly improved blood lipid and antioxidant levels. Moreover, microarray analysis of hepatic and adipose gene expression profiles revealed that TPP and TPS inhibited obesity through effects on the pathways of fatty acid biosynthesis, steroid hormone biosynthesis, unsaturated fatty acid biosynthesis, fatty acid elongation, glycerolipid metabolism, and glycerophospholipid metabolism.

**Conclusions:**

TPSM might be a potential therapy for the treatment of obesity.

The incidence of obesity has increased dramatically in recent years and has become a serious health medical problem in the world (1). Epidemiological studies have shown that obesity is a complex metabolic disorder caused by the imbalance between energy intake and energy expenditure and boosts the risk of various chronic diseases, such as hypertension, type II diabetes, coronary artery disease, and cancers (2, 3). Currently, the available therapy for obesity causes a number of side effects (4–6). Therefore, the prevention of obesity is an urgent matter, and the use of food to suppress the accumulation of body weight and body fat may be a safe solution (7, 8).

As a traditional type of Chinese tea (Camellia sinensis), oolong tea has been a popular beverage worldwide because of its health benefits including anti-oxidation, anti-microbial, cholesterol-lowering effect, and reducing the risks of cancer (9–11). Oolong tea polysaccharide, caffeine, and polyphenols contribute to these health benefits (12, 13). The polyphenols found in semi-fermented oolong tea mainly comprise epigallocatechin, epigallocatechin gallate (EGCG), epicatechin, and epicatechin gallate (14–16). Given the ever-growing obesity pandemic, the anti-obesity effects of oolong tea have been the focus of considerable attention (17). However, the strength of such anti-obesity effects differs depending on the variety and functional components. Rumpler et al. observed that oolong tea extract intake significantly increased the energy expenditure in a group of young males (18). Since then, clinical trials have reported the effects of tea preparations on increasing energy expenditure, fat oxidation, weight loss, fat mass, and weight maintenance after weight loss (19–21). Oolong tea extracts decreased weight and body fat gain in rodent models in several studies (18, 22). Kuo et al. reported that body weight suppression occurs in tea-leaf-fed groups in the following order: oolong tea > pu-erh tea > black tea > green tea (23).

Several studies have evaluated the effects of oolong tea extracts on obesity (24–26). However, only few studies have focused on the anti-obesity effect of specific components of oolong tea. Therefore, this study aimed to isolate polyphenols and polysaccharides from oolong tea and investigated their influence on the development of obesity.

## Materials and methods

### Chemicals and animals

The coarse old oolong tea employed in the study was produced in Fujian, China. A total of 145 male Sprague–Dawley rats (~200 g) were purchased from PLA Military Academy of Medical Sciences Laboratory Animal Center (license number SCXK [Beijing] 2012–0004).

### Extraction of tea polyphenols and polysaccharides from oolong tea

Crude oolong tea leaves (JinFuDe, Fujian Province, China) were ground into powder by a grinder. Water extracts were obtained three times at 85°C at a solid–liquid ratio of 1:15 once an hour. The extracts were filtered using a nine-layer gauze and concentrated under reduced pressure. One part of the concentrate was lyophilized as the total water extract. The other part of the concentrate was extracted by chloroform, and the aqueous layer was extracted with ethyl acetate. Finally, the organic layer was lyophilized to tea polyphenols (TPP), and the aqueous layer was precipitated with ethanol and lyophilized to tea polysaccharides (TPS) after protein removal. The obtained oolong TPP was analyzed through high-performance liquid chromatography (HPLC; Shimadzu, Kyoto, Japan) equipped with an ultraviolet (UV) detector. The oolong TPP is composed of EGC (59.09%), EGCG (12.83), EC (13.47%), and ECG (14.61%). The oolong TPS comprised 4.06 mol% rhammose, 2.58 mol% fucose, 27.19 mol% arabinose, 1.26 mol% xylose, 5.68 mol% mannose, 4.66 mol% galactose, and 54.57 mol% glucose.

### Animals and experimental protocols

All of the experimental procedures employed in the study followed the guidelines of the Committee on the Ethics of Animal Experiments of Tianjin University of Science and Technology (TUST20131015) and were based on the National Institutes of Health Guide for the Care and Use of Laboratory Animals. All efforts were exerted to minimize suffering.

Rats were kept in a room maintained under standard conditions of 20°C to 25°C, 12 h/12 h light/dark cycle, and relative humidity of 45 to 65%. After 7 days of adaptation, the rats were randomly divided into two groups (Group I = 10 and Group II = 135) by weight. Rats in Group I were given free access to distilled water and normal diet. Group II rats were orally administered with distilled water and a high-fat diet consisting of 79% basal feed, 10% lard, 10% egg yolk powder, 0.5% cholesterol, and 0.5% cholate. After a 2-week high-fat diet, the Group II rats were sorted by weight gain, and one-third of the rats with a slower speed of gaining weight (obesity-resistant rats, body weight gain less than 20%) were eliminated from the study. The 90 screened out obesity-sensitive rats were randomly divided into different groups as follows: model control, the group given 40 mg/kg orlistat, the group given 400 or 800 mg/kg total water extract, the group given 400 or 800 mg/kg TPP, the group given 400 or 800 mg/kg TPS, and the group given a combination of TPS and TPP at 400 mg/kg. All rats used in the experiment were given oolong tea functional materials via oral gavage. These groups were given high-fat diet and designated as normal control group (NC), model control group (MC), positive control group, orlistat (OC), high dose of total water extract group (TWH), low dose of total water extract group (TWL), high dose of tea polyphenols group (TPPH), low dose of tea polyphenols group (TPPL), high dose of tea polysaccharides group (TPSH), low dose of tea polysaccharides group (TPSL), and the complex of tea polysaccharides and polyphenols group (TPSM), respectively. During the experiment, food intake was recorded daily, and body weight and length were measured once every 3 days.

After 6 weeks of experimentation, all rats were fasted overnight, blood sample was collected from the abdominal femoral arteries of the rats, and the rats were sacrificed by cervical vertebral dislocation. The liver, heart, kidney, perinephric fat pads, epididymal adipose tissues, and mesenteric adipose tissues were quickly removed and weighed. Then, the tissues were stored at −80°C.

## Blood and liver biochemical analysis

The rat serum parameters, including triglyceride (TG), total cholesterol (TC), high-density lipoprotein cholesterol (HDLC), low-density lipoprotein cholesterol (LDLC), malondialdehyde (MDA) levels, and total superoxide dismutase (T-SOD) activities, were determined using commercially available kits (BioSino, Beijing, China). The serum leptin was characterized by immunoassay using rat/mouse ELISA kit (R&D Systems, Minneapolis, USA). Hepatic TG, TC, MDA, and T-SOD concentrations were estimated using the same kit for serum analysis. All measurements performed were in accordance with the manufacturers’ instructions.

### Adipose and liver histopathology

Suitable rat epididymal adipose and hepatic tissues were selected and fixed in 10% formalin for 16 h. Then, all of the tissues were dehydrated in graded ethanol (70% ethanol for 10 min, 80% ethanol for 10 min, 95% ethanol for 10 min thrice, and 100% ethanol for 15 min thrice). Xylene was used to clear the tissues (15 min twice). Then, the tissues were dipped in wax twice at 60°C, 1 h to 2 h each time, and paraffin embedded at the same temperature. Fat and liver tissue blocks were cut into five microsections and stained with hematoxylin and eosin.

### Gene chip analysis, reverse transcription polymerase chain reaction (RT-PCR) verification, and metabolic pathway analysis

Differentially expressed genes were screened for the NC, TPP, TPS, and TPSM groups using the gene expression profile array (GeneChip Rat Genome 230 2.0 Array, Affymetrix, Santa Clara, CA, USA). In addition, the differentially expressed genes screened by gene chip arrays were verified through RT-PCR. Specific primers for the genes were designed, synthesized, and diluted with sterile water in accordance with the manufacturer’s manual. The total RNA in the liver and epididymal adipose tissues served as raw material, whereas the rat Glyceraldehyde-3-phosphate dehydrogenase (GADPH) genes were used as housekeeping genes. The One-Step SYBR PrimeScript PLUS RT-PCR Kit (TaKaRa, Shiga, Japan) was used for PCR. The differentially expressed genes screened through gene chip arrays and verified via RT-PCR were used to determine the related metabolic pathways with the GenBank ID in the national center for biotechnology information (NCBI) gene database and Kyoto Encyclopedia of Genes and Genomes (KEGG) metabolic pathway database. The effects of the resulting genes on the fat metabolic pathway were also explored.

### Statistical analysis

The results are presented as means with their standard errors. Statistical analysis was performed using the Statistical Product and Service Solutions (SPSS) program. Data were analyzed by one-way analysis of variance (ANOVA). Differences between the groups were established using the least significant difference test, and the criterion for statistical significance was set at *p* < 0.05.

## Results

### Changes in rat body weight, food utilization, and Lee’s index

Table 1 shows the changes in body weight, food utilization (body weight gain/food intake × 100%), and Lee’s index (3√body weight × 1000/body length). The final body weight of rats in the MC group was significantly higher than that of the NC group. By contrast, all of the treated groups, except for TPSL, exhibited a significant reduction in body weight gain compared with the MC group. Moreover, TPSM group showed the highest anti-obesity effects when compared with the other groups. These results indicate that polysaccharides and polyphenols were synergistic in reducing body weight gain. Meanwhile, all of the groups, except for TPSH, exhibited a significant decrease in Lee’s index compared with the MC group.

**Table 1:** Body weight and other characters of rats in each group

Groups	Initial BW(g)	Final BW(g)	Lee’s index	Food utilization (%)
NC	228.58 ± 14.03	459.31 ± 27.19	311.21 ± 11.83	18.73% ± 2.02%
MC	348.74 ± 22.35	548.74 ± 51.74	339.55 ± 8.06	26.87% ± 0.98%
OC	351.33 ± 17.90	511.86 ± 43.54**#	322.31 ± 9.66**##	24.35% ± 1.76%
TWH	346.51 ± 19.37**	466.78 ± 34.21##&&	316.08 ± 9.64##	19.78% ± 2.16%#
TWL	352.16 ± 21.12**	478.61 ± 45.36##	318.45 ± 12.83##	20.56% ± 2.29%#
TPPH	348.44 ± 16.09**	473.26 ± 32.75##&	315.34 ± 5.85##	20.37% ± 3.12%#
TPPL	347.89 ± 13.16**	485.57 ± 34.27##	324.27 ± 9.03**##	21.51% ± 2.29%#
TPSH	349.89 ± 18.27**	522.72 ± 42.43**	328.56 ± 5.68**#	22.67% ± 2.03%
TPSL	343.06 ± 16.62**	505.92 ± 32.48**#	325.73 ± 7.79**##	21.67% ± 1.97%#
TPSM	345.73 ± 12.75**	447.67 ± 33.80##&&	318.49 ± 10.14##	19.04% ± 2.14%#

All values are means ± SD (*n* = 10). Values with different superscripts are significantly different among the groups by ANOVA with Duncan’s multiple range test from NC at * *P* < 0.05,

** *P* < 0.01; MC at

# *P* < 0.05,

## *P* < 0.01; OC,

& *P* < 0.05,

&& *P* < 0.01.

### Body fat weight

At the end of the experiment, the perinephric fat pads, epididymal adipose tissues, and mesenteric adipose tissues of each group were collected and measured. MC group exhibited a significantly higher weight of the three fat parts than that of the NC group. Meanwhile, tea water extract, TPP, TPS, and TPSM decreased in adipose tissue.

## Blood and liver biochemical profiles

**Fig. 1:**
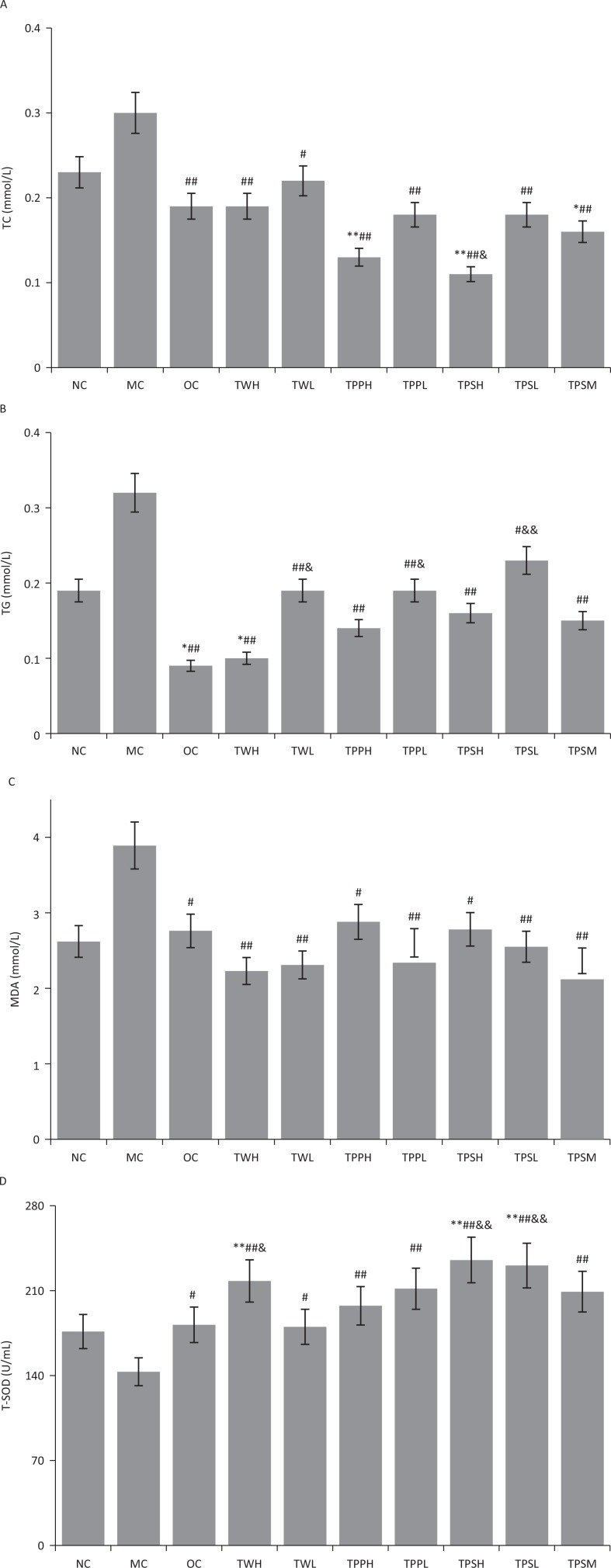
The effect of oolong tea extracts on rat hepatic lipid profile and antioxidants. (a) Hepatic contents of TG and TC. (b) Hepatic contents of TG and TC. (c) Hepatic MDA content. (d) Hepatic total SOD content. Values with different superscripts are significantly different among the groups by ANOVA with Duncan's multiple range test from NC at *P < 0.05, **P < 0.01; MC at ^#^*P* < 0.05, ##*P* < 0.01; OC, ^&^*P* < 0.05, ^&&^*P* < 0.01.

Hepatic lipid and antioxidant profiles are shown in Fig. 1 and Table 2. The triglyceride and cholesterol levels of rats in the MC group increased significantly compared with the NC group. Meanwhile, the serum triglyceride and cholesterol levels lowered significantly in the TWH, TPPH, TPPL, and TPSM groups. Moreover, the serum MDA and T-SOD levels reduced significantly in the TPSM, TWH, and TPPL groups; similar results were also observed in the hepatic MDA and T-SOD levels. However, the serum LDL-C levels in TWL, TPPH, TPPL, TPSH, TPSL, and TPSM groups were significantly lower than that of the MC group. TPSL and TPSM groups also exhibited significantly lower leptin levels than the other groups.

**Table 2:** Serum figures of rats in each group

Groups	TC (mmol/)	TG (mmol/L)	HDLC (mmol/L)	LDLC (mmol/L)	MDA (mmol/L)	T-SOD (U/mL)	LEP (pg/mL)
NC	1.57 ± 0.25	0.86 ± 0.42	2.22 ± 1.64	0.94 ± 0.23	0.12 ± 0.09	157.03 ± 20.24	179.73 ± 12.12
MC	2.58 ± 0.43	1.23 ± 0.26	1.14 ± 0.16	1.52 ± 0.56	0.27 ± 0.03	122.99 ± 17.16	198.82 ± 27.41*
OC	1.56 ± 0.32##	0.76 ± 0.26##	1.71 ± 0.55	0.40 ± 0.48	0.21 ± 0.15*	184.48 ± 15.27**##	178.82 ± 25.42#
TWH	1.99 ±0.43*##&	0.61 ± 0.28##	1.98 ± 0.53##	0.91 ± 0.31#&	0.15 ± 0.07##	187.06 ± 18.00**##	182.91 ± 22.67
TWL	1.80 ± 0.77##	0.64 ± 0.33##	1.74 ± 0.42#	0.67 ± 0.78##	0.13 ± 0.07##	154.45 ± 18.70##&&	185.64 ± 29.16
TPPH	1.97 ± 0.41*##&	0.77 ± 0.20##	2.26 ± 0.40##	0.73 ± 0.46##	0.17 ± 0.08##	97.82 ± 24.34**##&&	175.18 ± 19.78#
TPPL	1.75 ± 0.48##	0.68 ± 0.16##	1.75 ± 0.43##	0.43 ± 0.64*##	0.10 ± 0.04##&&	163.03 ± 18.09##&	178.67 ± 21.07#
TPSH	1.86 ± 0.43##	1.11 ± 0.34&	1.65 ± 0.31	0.67 ± 0.34##	0.17 ± 0.11#	174.83 ± 23.80*##	179.86 ± 16.80
TPSL	1.58 ± 0.27##	1.22 ± 0.53*&&	1.41 ± 0.34**	0.36 ± 0.78*##	0.21 ± 0.11*	192.21 ± 11.58**##	166.50 ± 15.71*##
TPSM	1.55 ± 0.25*##	0.69 ± 0.31##	1.73 ± 0.58#	0.27 ± 0.43**##	0.13 ± 0.04##&	146.94 ± 19.69#&&	170.50 ± 19.16##

All values are means ± SD (*n* = 10). Values with different superscripts are significantly different among the groups by ANOVA with Duncan’s multiple range test from NC at

* *P* < 0.05,

** *P* < 0.01; MC at

# *P* < 0.05,

## *P* < 0.01; OC,

& *P* < 0.05,

&& *P* < 0.01.

### Histological analysis of liver and epididymal white adipose tissue

Changes in the degree of infiltration of lipid droplets in the liver are shown in Supplementary Fig. 1. The representative liver section of the MC group showed increased infiltration of lipid droplets, leading to a hepatic steatotic condition. Meanwhile, the infiltration of lipid droplets in the representative liver section of the other groups was markedly reduced; even the liver histological section of the TPSM group was free from lipid droplets. The histology of the rat epididymal white adipose tissue is shown in Supplementary Fig. 2, and the numbers of adipocytes within the same field are presented in Supplementary Table 1. The adipocyte size of the MC group was significantly larger than that of the NC group, and the numbers of fat cells in the MC group were significantly less than that of the NC group. In all of the medicated groups, adipocyte sizes were significantly smaller, and the number of fat cells was significantly lower than those of the MC group.

### Result of cDNA microarray

The Affymetrix GeneChip^®^ Rat Genome 230 2.0 Array includes approximately 31,000 probe sets representing 28,000 specific genes. According to the results provided by the Tianjin Biochip Corporation, we screened out the differentially expressed genes related to lipid metabolism in the liver and epididymal adipose tissues. Under the premise of greater than twofold difference relative to that of the MC group, we detected 11 differentially co-expressed genes in the liver of the NC, TPPL, TPSL, and TPSM groups. Among the 11 genes, 2 genes were significantly upregulated and 9 genes were significantly downregulated. In the same circumstance, we detected 13 differentially co-expressed genes in the epididymal adipose tissues of the NC, TPPL, TPSL, and TPSM groups. Among the 13 genes, 6 genes were significantly upregulated and 7 genes were significantly downregulated (Table 3). Then, these genes were searched on the KEGG website to determine their lipid metabolism pathways (Supplementary Figs. 3–8). Microarray analysis revealed that TPP and TPS inhibited obesity through effects on the pathways of fatty acid biosynthesis, steroid hormone biosynthesis, unsaturated fatty acid biosynthesis, fatty acid elongation, glycerolipid metabolism, and glycerophospholipid metabolism.

**Table 3:** Co-expression of regulated genes related to lipid metabolism

Tissue	Probe set	Gene symbol	Gene title	RefSeq transcript ID	Levels
**Live tissue**	1367667_at	Fdps	Farnesyl diphosphate synthase	NM_031840	down
	1367707_at	Fasn	fatty acid synthase	NM_017332	down
	1387006_at	Sult2al1	Sulfotransferase family 2A, dehydroepiandrosterone (DHEA)-preferring-like 1	NM_012695	down
	1370281_at	Fabp5	Fatty acid binding protein 5, epidermal	NM_145878	down
	1370355_at	Scd1	Stearoyl-coenzyme A desaturase 1	NM_139192	down
	1387123_at	Cyp17a1	Cytochrome P450, family 17, subfamily a, polypeptide 1	NM_012753	down
	1387156_at	Hsd17b2	Hydroxysteroid (17-beta) Dehydrogenase 2	NM_024391	down
	1370566_at	Rdh2	Retinol dehydrogenase 2	NM_199208	down
	1374914_at	Ppard	Peroxisome proliferator-activated receptor delta	XM_001078083 ///	down
	1380013_at	Pnpla3Patatin-like phospholipase domain containing 3	XM_343302	up
	1388108_at	Elovl6	ELOVL family member 6, elongation of long chain fatty acids (yeast)	NM_134383 /// XM_001075165	up
**Adipose tissues**	1369179_a_at	Pparg	Peroxisome proliferator-activated receptor gamma	NM_001145366 /// NM_001145367 /// NM_013124	down
	1367585_a_at	Atplal	ATPase, Na+/K+ transporting, alpha 1 polypeptide	NM_012504	down
	1367609_at	Mif	Macrophage migration inhibitory factor	NM_031051	down
	1367894_at	Insig1	Insulin induced gene 1	NM_022392	down
	1370862_at	Apoe	Apolipoprotein E	J02583	down
	1371691_at	Rarres2	Retinoic acid receptor responder (tazarotene induced) 2	BI282993	down
	1371963_at	Pcca	Propionyl-coenzyme A carboxylase, alpha polypeptide	BF395042	down
	1375034_at	Pla2g15	Phospholipase A2, group XV	AI410383	up
	1387132_at	Lipe	Lipase, hormone sensitive	NM_012859	up
	1387365_at	Nr1h3	Nuclear receptor subfamily 1, group H, member 3	NM_031627	up
	1379075_at	Mboat2	Membrane-bound O-acyltransferase domain containing 2	AI501287	up
	1382680_at	Adfp	Adipose differentiation-related protein	BG673602	up
	1386960_at	Slc37a4	Solute carrier family 37 (glucose-6-phosphate transporter), member 4	NM_031589	up

## Discussion

With the dramatically increasing prevalence of obesity, the urgent need for new strategies to combat the growing epidemic emerges. Therefore, in the present study, we isolated polyphenols and polysaccharides from oolong tea and investigated the anti-obesity capabilities of the components. In the present study, TWH, TWL, TPPH, TPPL, and TPSM effectively prevented rat body weight increase relative to the weight of the MC group. Moreover, the tea water extract and polyphenols presented dose-dependent effects; the high-dose green tea water extract or polysaccharide was more efficacious in preventing obesity than the other treatments tested. Nevertheless, TPSH and TPSL did not significantly change the rat body weight.

He et al. reported that oolong tea could reduce the weight of rats by suppressing food intake (27). Kao et al. also observed a reduction in food intake after the administration of EGCG present in TPP (26). We also observed a decrease in weight gain induced by polyphenols, polysaccharide, or the combination of TPS and TPP, which is relevant in food utilization.

Xu et al. reported that green tea extract, polysaccharide, and polyphenols exhibit anti-inflammatory activities (28). Our results indicated that polyphenols, polysaccharide, or the combination of TPS and TPP can significantly reduce serum and liver triglyceride levels. Furthermore, the TPSL group effectively increased serum T-SOD levels.

Numerous studies on genetic, metabolic, hormonal, behavioral, social, and cultural aspects have been conducted to increase our understanding of the cause and treatment of obesity (29, 30). The physiological and molecular changes observed in our high-fat-diet-induced obese rat model provided useful insight into the development of obesity in humans. In the present study, we showed that the expression of a number of genes involved in lipid metabolism was altered in the MC group, which further demonstrated the usefulness of this high-fat diet model. In addition, a total of 24 obesity-related genes showed significant changes in liver and epididymal adipose tissues in the TPPL, TPSL, and TPSM groups compared with the MC group. Considerable evidence from the KEGG website reveals that these genes play important roles in the pathogenesis of obesity. The pathways participated in by the genes include fatty acid biosynthesis, steroid hormone biosynthesis, unsaturated fatty acid biosynthesis, fatty acid elongation, glycerolipid metabolism, and glycerophospholipid metabolism.

## Conclusions

In summary, crude old oolong tea extract prevented body weight gain in male Sprague–Dawley rats, in which polyphenols and polysaccharide may play an important role, particularly the combination of polyphenols and polysaccharide. Multiple factors in oolong tea were determined to contribute to the anti-obesity effects in the present research. Each main ingredient in oolong tea contributed to the anti-obesity function, and every ingredient has potential beneficial effects in achieving weight loss, such as reducing food utilization, lowering serum triglyceride levels, inhibiting fatty acid absorption, and regulating relevant gene expression. Therefore, the combination of polysaccharide and polyphenols might be a potential therapy to treat obesity, and further clinical studies are needed in this regard.

## Supplementary Material

Oolong tea polysaccharide and polyphenols prevent obesity development in Sprague–Dawley rats
