# Supplementary material for: Oolong tea polysaccharide and polyphenols prevent obesity development in Sprague–Dawley rats
Source: Food Nutr Res. 2018 Dec 19;62:10.29219/fnr.v62.1599. doi: 10.29219/fnr.v62.1599 (PMC6303733; doi:10.29219/fnr.v62.1599)

**SUPPLEMENTARY MATERIAL**

**Oolong tea polysaccharide and polyphenols prevent obesity development in Sprague–Dawley rats**

*Tao Wu^1,2,3^, Jinling Xu^1^, Yijun Chen^1,2^, Rui Liu^1,2^,Min Zhang^1,2,^*^*^

*^1^* *Beijing Advanced Innovation Center for Food Nutrition and Human Health, Beijing Technology & Business University, Beijing, China*

*^2^State Key Laboratory of Food Nutrition and Safety (Tianjin University of Science and Technology), Ministry of Education, Tianjin 300457, China*

*^3^Tianjin Food Safety & Low Carbon Manufacturing Collaborative Innovation Center, 300457, Tianjin, China*

*The first two authors contributed to the work equally and should be regarded as co-first authors.*

*Corresponding to: Min Zhang, Professor, Tianjin University of Science and Technology.

Tel. /fax: +86 22 60912430

*E-mail address*: [zm0102@tust.edu.cn](mailto:zm0102@tust.edu.cn)

**Table S 1 Body fat figures of rats in each group**

| **Groups** | **Fat weight(g)** | **Fat index (%)** | **Number of cell** |
| --- | --- | --- | --- |
| **NC** | 19.43±5.81 | 4.30±1.25 | 186.35±14.43 |
| **MC** | 38.96±10.97 | 6.78±1.38 | 98.23±7.05 |
| **OC** | 30.89±6.73^**#^ | 5.98±1.12^**^ | 117.20±10.68^**##^ |
| **TWH** | 22.19±4.56^##&^ | 4.58±0.79^##&^ | 131.70±21.90^##^ |
| **TWL** | 26.05±11.47^##^ | 5.17±1.68^##^ | 122.80±28.29^**##^ |
| **TPPH** | 23.98±6.52^##^ | 4.95±1.08^##^ | 143.75±12.35^##^ |
| **TPPL** | 28.16±8.21^*##^ | 5.48±1.41^*#^ | 129.83±18.88^**##^ |
| **TPSH** | 35.31±9.28^**^ | 6.45±1.33^**^ | 142.55±32.90^##^ |
| **TPSL** | 33.69±10.18^**^ | 6.39±1.54^**^ | 177.26±25.92^##^ |
| **TPSM** | 22.78±4.83^##&^ | 4.94±0.88^##^ | 199.18±30.25^##^ |

All values are means ± SD (n = 10). Values with different superscripts are significantly different among the groups by ANOVA with Duncan's multiple range test at P < 0.05. *：NC，P<0.05，**：P<0.01；^#^ MC，P<0.05，^##^：P<0.01；^&^：OC，P<0.05，^&&^：P<0.01。

**Figure** **S1 Histopathological analysis of liver.**


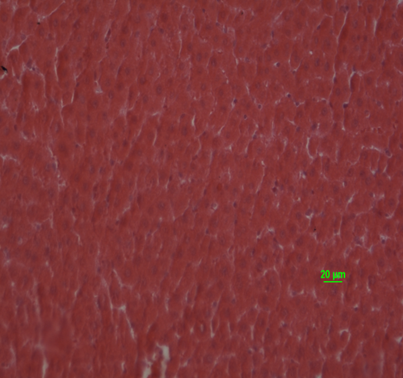

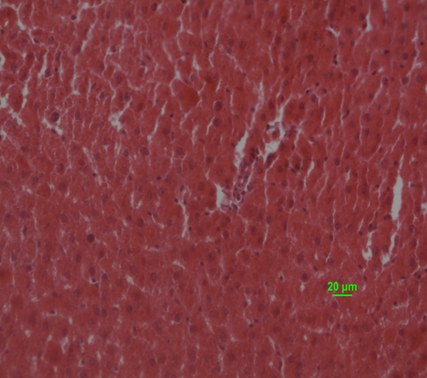

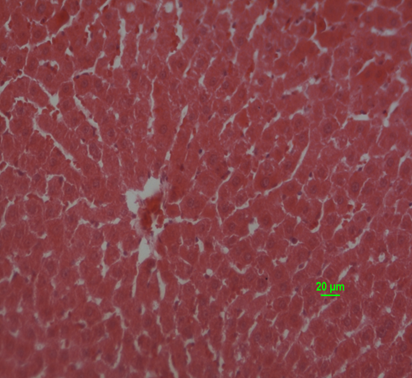

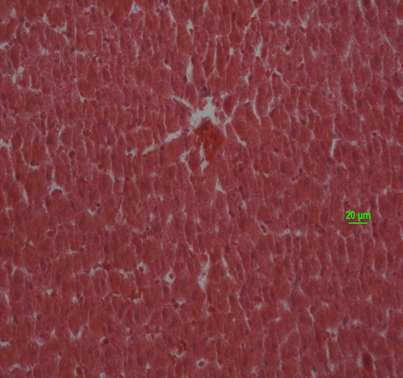

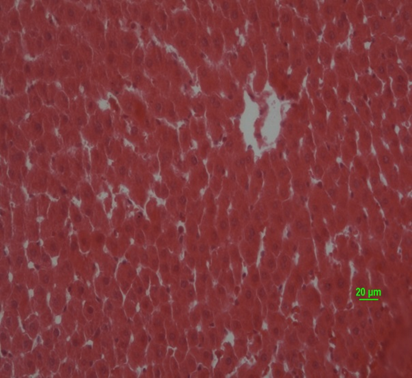


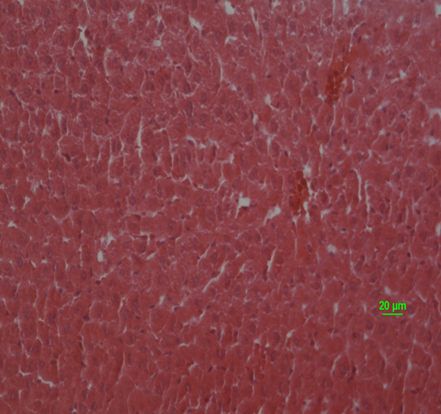

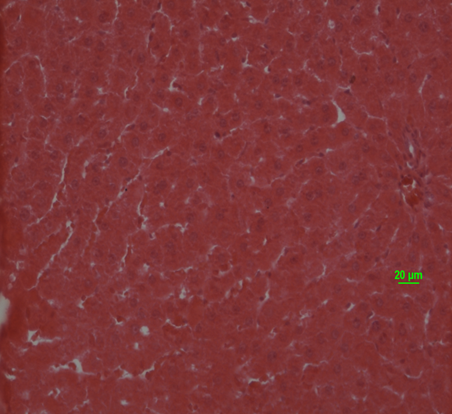

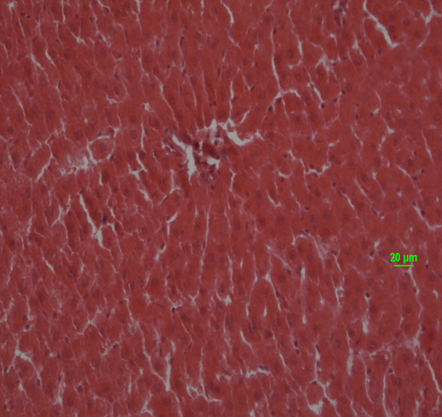

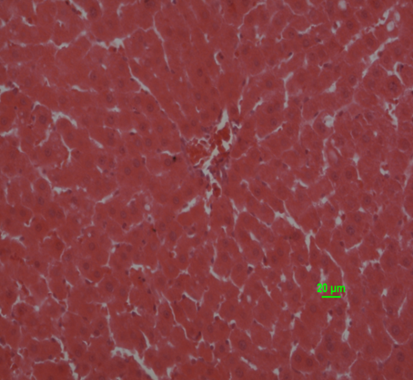

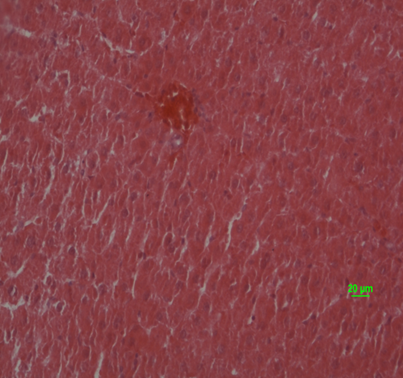


**NC MC OC TWH TWL**

**TPPH TPPL TPSH TPSL TPSM**

**Figure** **S2 Histopathological analysis of adipose tissues.**


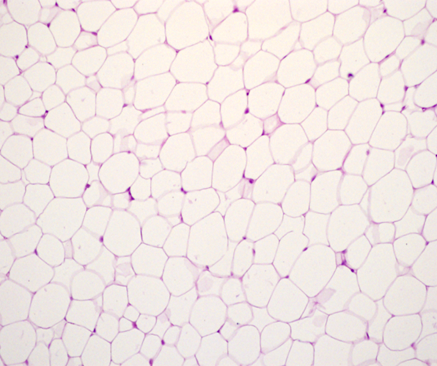

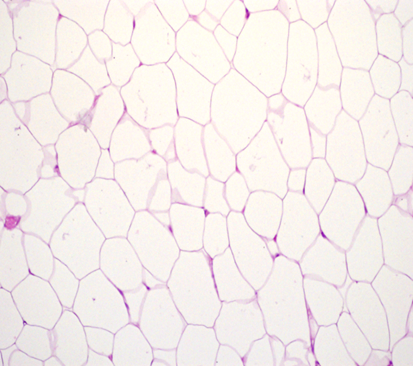

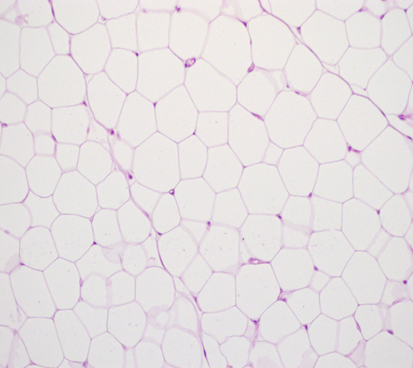

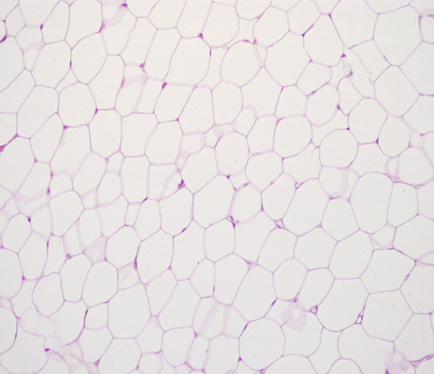

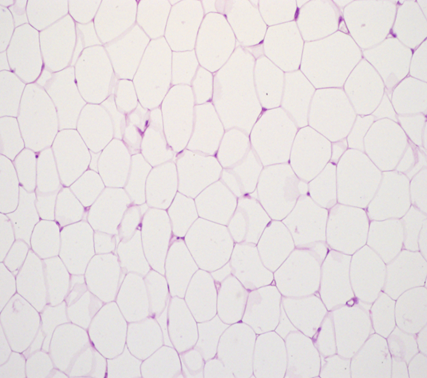


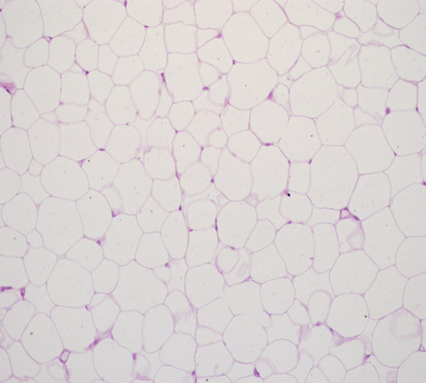

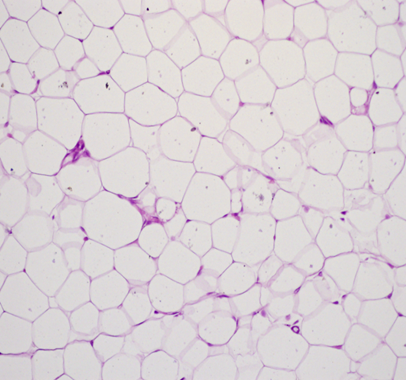

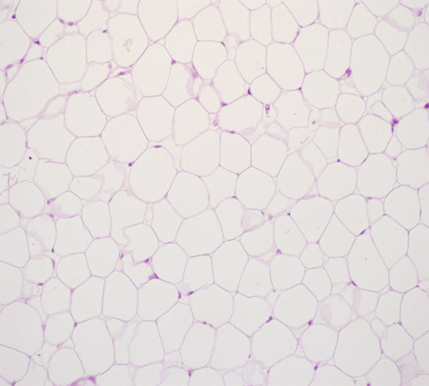

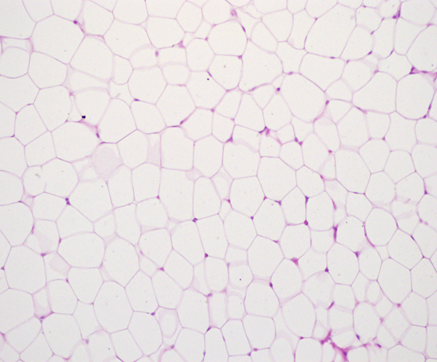

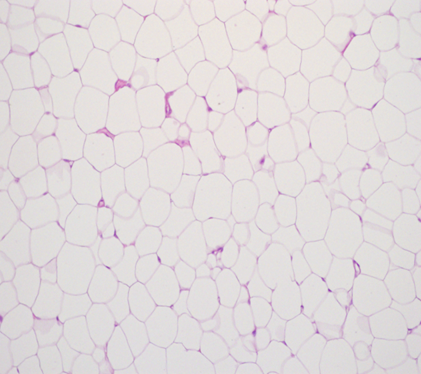


**NC MC OC TWH TWL**

**TPPH TPPL TPSH TPSL TPSM**

**Figure** **S3. Fatty acid biosynthesis pathway**


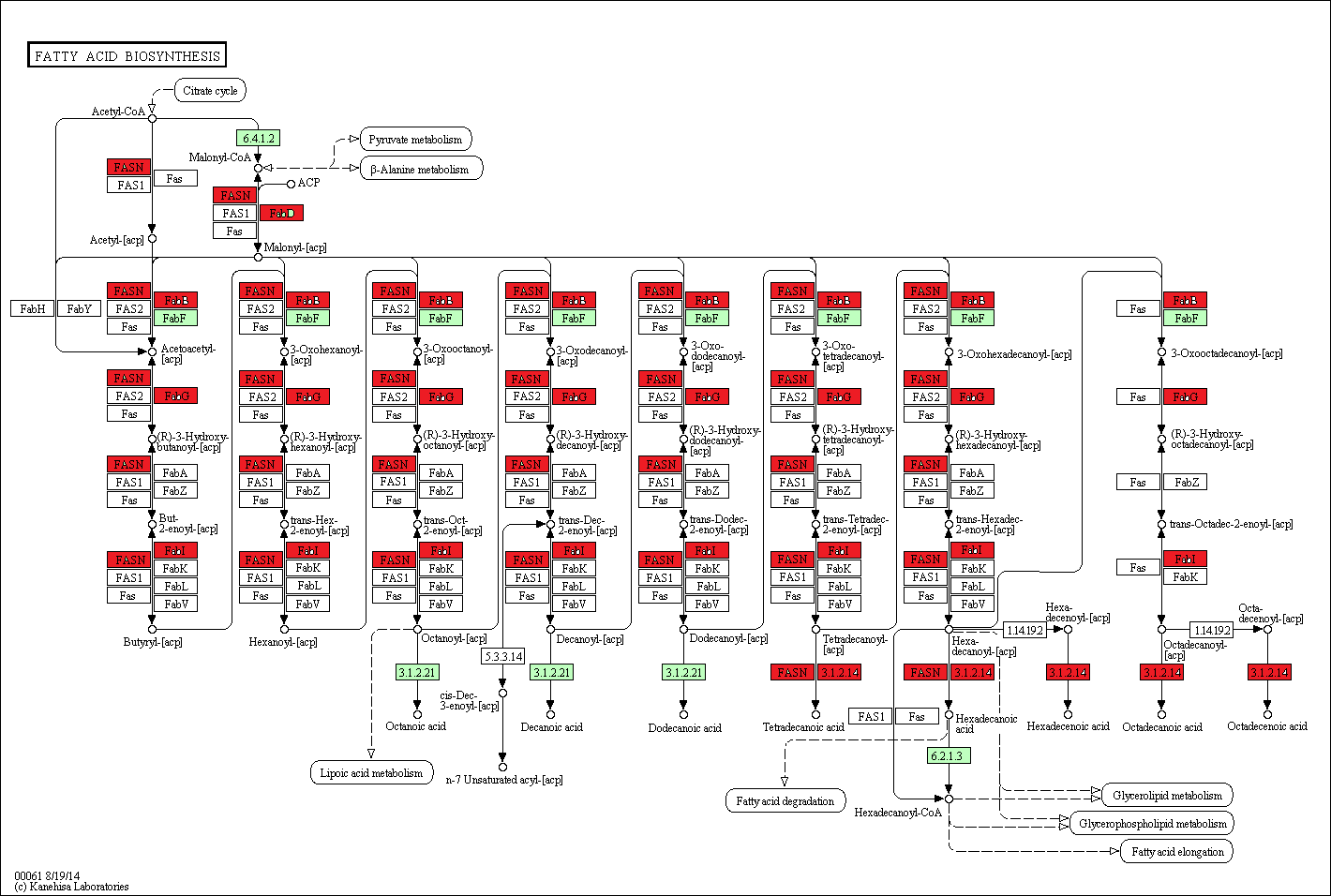


**Figure** **S4 Steroid hormone biosynthesis pathway**


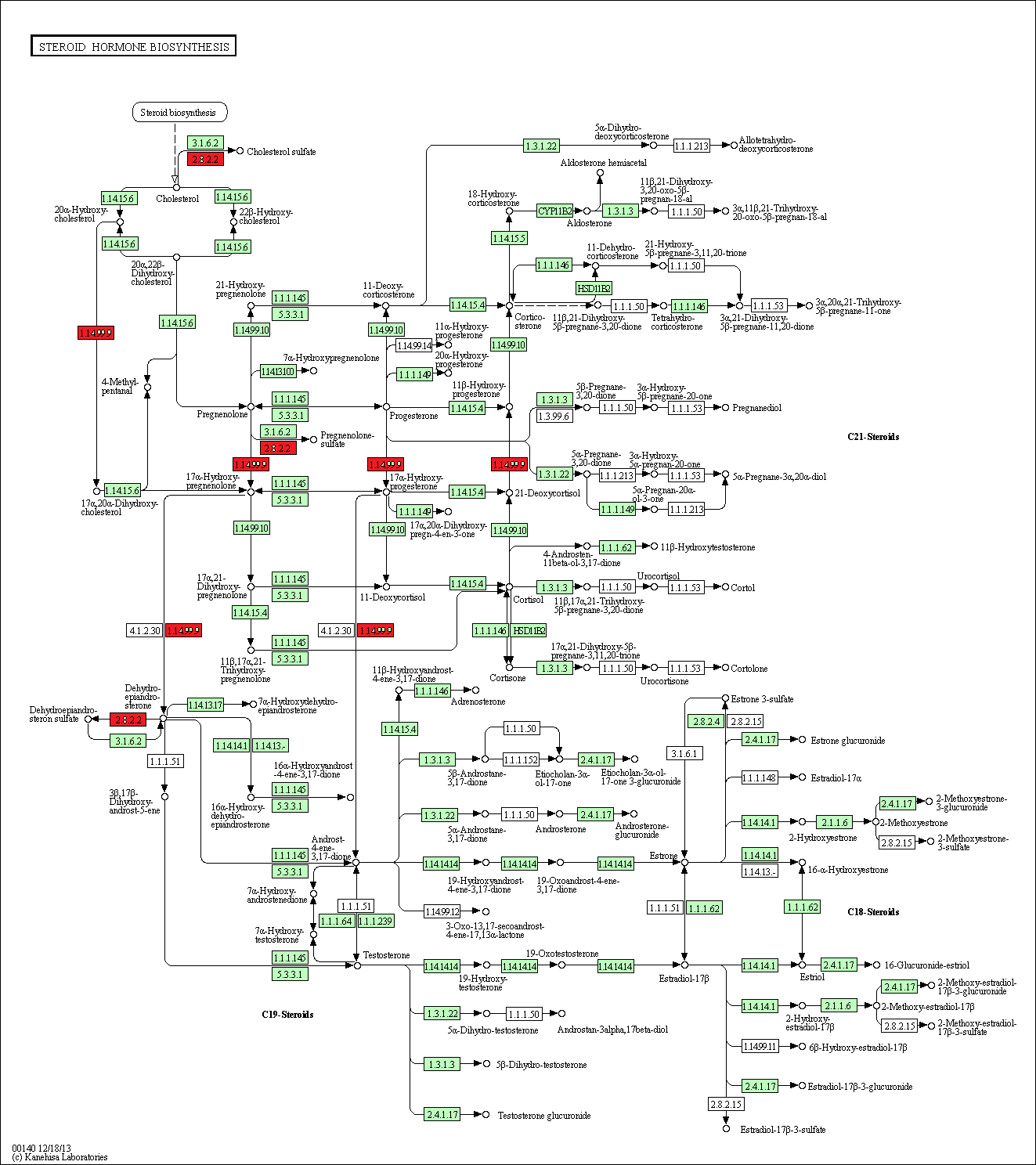


**Figure** **S5 Biosynthesis of unsaturated fatty acids pathway**


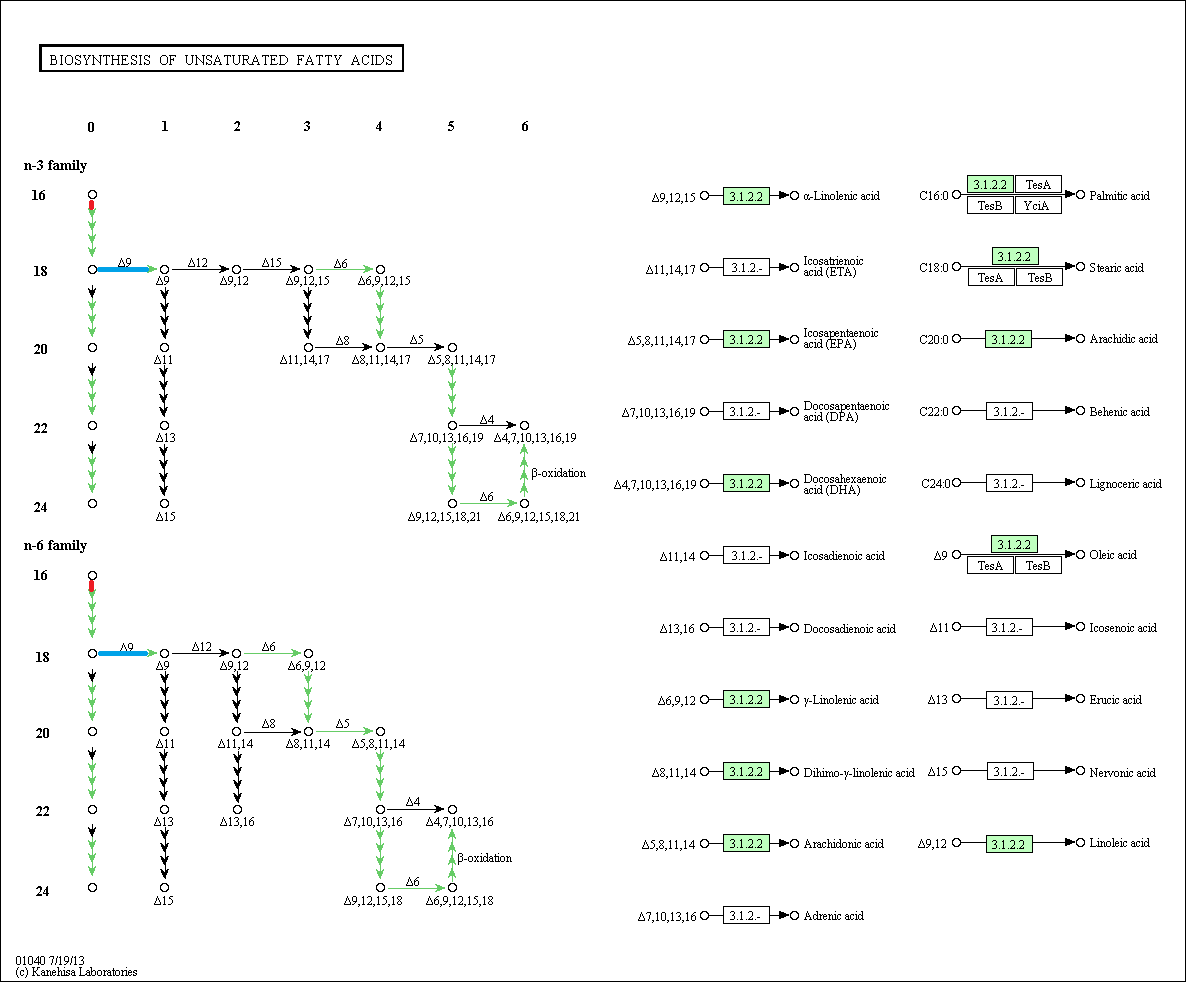


**Figure** **S6 .Fatty acid elongation pathway**


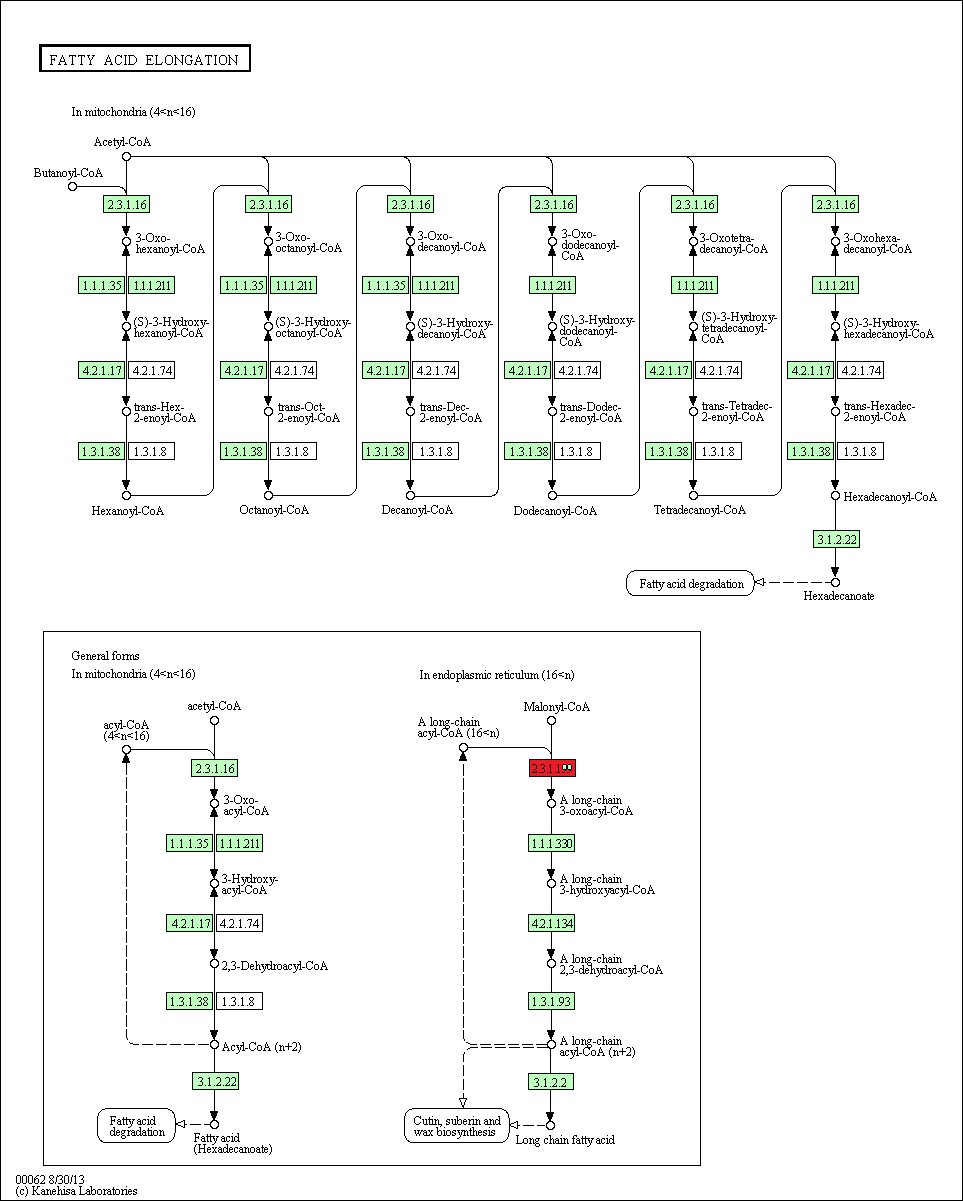


**Figure** **S7 Glycerolipid metabolism pathway**


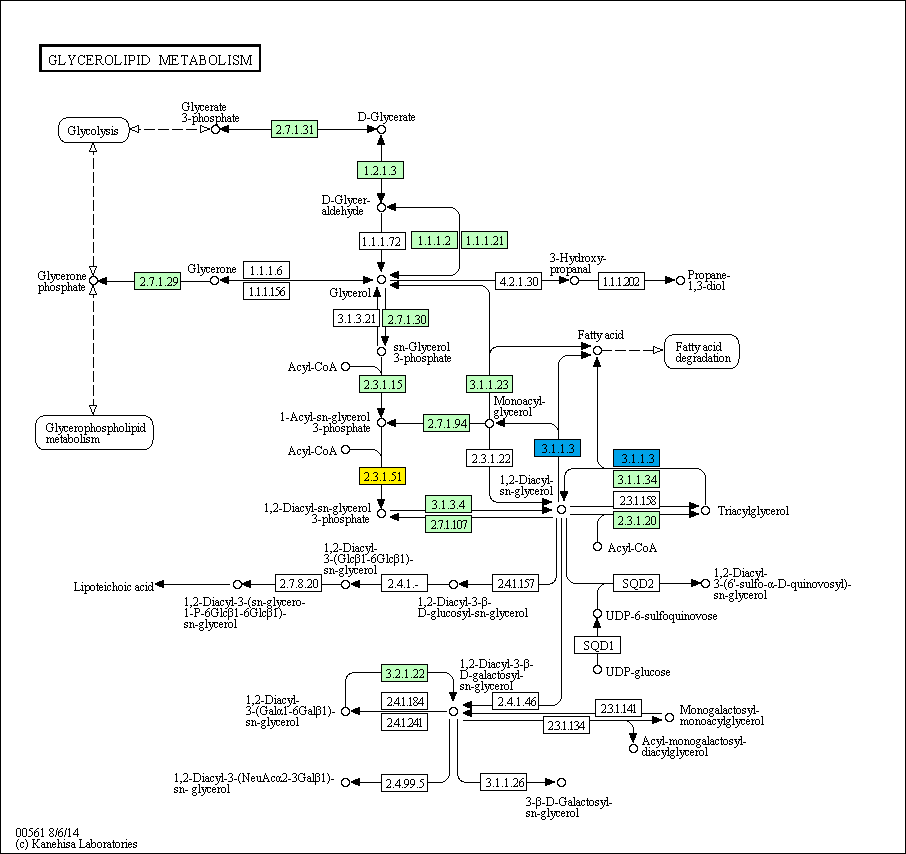

**Figure** **S8. The effect of Oolong tea on glycerophospholipid metabolism.**


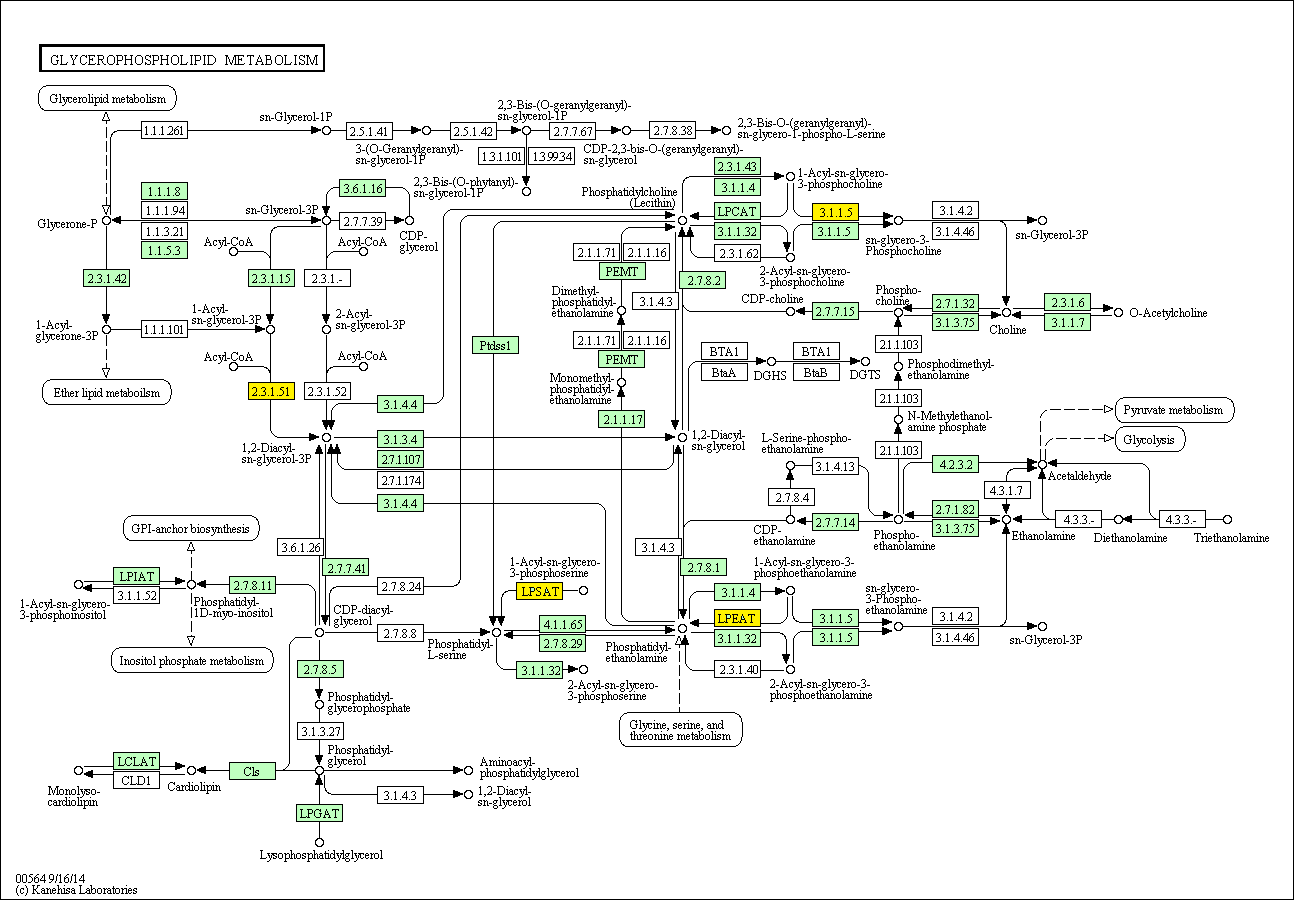

Supplement: Oolong tea polysaccharide and polyphenols prevent obesity development in Sprague–Dawley rats [file FNR-62-1599-s001.docx]
